# Promoting physical activity to patients: a scoping review of the perceptions of doctors in the United Kingdom

**DOI:** 10.1186/s13643-023-02245-x

**Published:** 2023-06-24

**Authors:** Gemma Woodhead, Divya Sivaramakrishnan, Graham Baker

**Affiliations:** 1 Edinburgh Medical School, University of Edinburgh, Scotland, UK; 2 Scottish Collaboration for Public Health Research and Policy, School of Health in Social Sciences, University of Edinburgh, Scotland, UK; 3 Physical Activity for Health Research Centre, Moray House School of Education & Sport, University of Edinburgh, Scotland, UK

## Abstract

**Background:**

The physician–patient encounter presents an ideal opportunity for physical activity (PA) promotion. This review aims to (i) explore the breadth and depth of existing literature investigating doctors’ perceptions of PA promotion in the United Kingdom (UK) and (ii) identify factors influencing the extent to which doctors engage in PA promotion during patient interactions.

**Methods:**

A five-stage scoping review methodology and the PRISMA-ScR guidance were followed: Stage 1—research questions specified; Stage 2—relevant studies identified by searching five electronic databases and manual screening of references; Stage 3—studies screened using Covidence™; Stage 4—study data extracted and charted; and Stage 5—findings from included studies were analysed, summarised and reported using (i) descriptive numerical analysis to provide insight into study characteristics and (ii) narrative summary of the evidence categorised by factors that influence doctors’ engagement with PA promotion.

**Results:**

In total, 16,961 studies were screened. Nineteen studies were included in the review with most conducted in primary care focusing on the perceptions of general practitioners. Seven influencing factors were identified: knowledge and training, personal interest and PA level, time, resources, confidence, the perceived role of the doctor and patient relevance.

**Conclusion:**

This review provides new evidence that historical barriers and influencing factors have a persisting impact on the ability and willingness of UK doctors to engage with PA promotion with patients. Previous efforts to address these factors would appear to have had limited success. Further intervention efforts are required to ensure more widespread and effective PA promotion to patients.

**Supplementary Information:**

The online version contains supplementary material available at 10.1186/s13643-023-02245-x.

## Key points

- Doctors in the United Kingdom face many challenges to promoting physical activity to their patients such as time, limited knowledge and a lack of confidence.
- Several of these challenges are historical, suggesting that previous attempts to change the behaviour of doctors have had limited success.
- Interventions targeting doctors should provide education, training and examples of good practice through modelling to promote more widespread promotion of physical activity to patients.

## Introduction

The health benefits of regular physical activity (PA) are well documented and wide ranging; almost everyone can benefit from being more active [1]. The improvements on physical [1], psychological and cognitive wellbeing [2] are undeniable, yet data published in 2020 showed that in the United Kingdom (UK), only between 56 and 67% of adults were active as per government guidelines [3–5]. This physical inactivity costs the UK National Health Service (NHS) an estimated £1.06 billion per year [6] and is responsible for one in six UK deaths [7].

A large body of evidence implicates societal, cultural, economic and environmental shifts in the decline of PA over recent decades [8]. Therefore, a whole-system approach, encompassing and acknowledging all such factors to increase the population’s activity across multiple settings is advocated [9]. One important setting which may help to create active societies is that of healthcare systems [9]. The Royal College of General Practice in the UK identified PA as a clinical priority in 2016 [10], and there are calls from the medical community to view PA as the ‘fifth vital sign’ [11]. The physician–patient encounter presents an ideal opportunity for PA promotion, with 78% of the population visiting their general practitioner (GP) each year [12], and growing evidence supports the effectiveness of PA interventions in primary care [13].

In the UK, age-specific PA guidelines are readily available for physicians to access and apply during consultations [14]. In 2013, the National Institute for Clinical Excellence (NICE) published recommendations and guidance for doctors delivering PA counselling, including information on ‘brief PA advice’, the simple ‘ask-assess-advise’ approach and use of screening tools, e.g., the general practice PA questionnaire [6]. Alternative approaches include motivational interviewing or a more formal exercise prescription [15] using the frequency, intensity, time, type (FITT) format. ‘Moving Medicine’ is an initiative by the Faculty of Sport and Exercise Medicine (FSEM) UK aiming to provide clinicians with accessible, evidence-based guidance on PA promotion based on the patient’s medical conditions [16]. Although these various PA promotion approaches exist, no particular framework or technique is endorsed, leaving this decision up to the physician depending on the nature and context of the patient encounter.

Despite the growth of favourable evidence of the benefits of PA, and a consensus that it is appropriate for healthcare professionals (HCPs) to promote PA to patients [17, 18], research demonstrates many physicians fail to do so. For example, one study found that 72% (of 1013 UK GPs) do not discuss PA with patients [19]. Global data on the perceptions of HCPs promoting PA to patients have identified enablers to PA promotion including practitioners’ own knowledge and skills [17, 18, 20] and positive attitudes towards PA promotion [20]. Notable barriers include insufficient time [17, 18, 21–25], lack of formal education or training [17, 19, 23–26], competing priorities [24, 26] and a perception that patients lack motivation to be active [20, 25, 26].

Although existing reviews of studies from across the world provide insight into perceptions, the strength of these reviews are limited by dated primary data [24, 27], inclusion criteria meaning heterogenous study contexts [18] and the involvement of different HCPs [25]. The training that different types of HCPs receive, as well as the guidelines to which they adhere, vary depending on the profession, presenting a challenge when interpreting study findings. In addition, different geographical locations of studies lead to uncertainty regarding the relevance of these studies to a UK context. Furthermore, updates to UK PA guidelines mean that findings from older studies (pre-2011) may be less relevant to current clinical practice. Therefore, a focused examination of recent UK-based evidence concentrating solely on doctors based in both primary and secondary care is timely and would ensure relevance to current UK practice. Therefore, the aims of this study were three-fold: (i) to establish the breadth and depth of research examining the perceptions of UK doctors on promoting PA to patients, (ii) to identify the factors influencing their promotion of PA and (iii) to determine what gaps in the research exist.

## Methods

### Study design

A scoping review was determined to be the most appropriate research method given the primary aim of this study was to provide a comprehensive overview of current research, whilst also allowing for the identification of key barriers and enablers to promotion and finally the identification of research gaps [28]. There were uncertainties about the extent to which doctor specific data could be extracted from studies involving multiple health professionals; thus, we viewed the literature base as complex and heterogenous, which may not ultimately be explanatory in nature. Therefore, we determined a scoping review to be more appropriate than a mixed methods systematic review at the outset. The protocol was drafted using the five-stage scoping review methodological framework proposed by Arksey and O’Malley [28] and Levac et al. [29] and was an iterative process, while following the Preferred Reporting Items for Systematic Reviews and Meta-Analyses extension for scoping reviews guidance (PRISMA-ScR) [30].

### Stage 1: Identify research questions

This research aimed to provide an overview of the perceptions of UK doctors on PA promotion in a healthcare setting. To address this, three research questions were generated:

1. What research exists examining the perceptions of UK doctors on PA promotion?
2. What factors influence PA promotion by UK doctors?
3. What are the gaps in the research regarding perceptions of UK doctors on PA promotion?

### Stage 2: Identify relevant studies

Relevant studies were identified by the following:

1. Electronic database searching using SCOPUS, Ovid (MEDLINE), Ovid (EMBASE), Cochrane library and Psychnet
2. Manually searching reference lists of key studies (i.e., relevant previous reviews and primary studies)

The varied terminology of PA promotion and perceptions required a comprehensive database search strategy. The initial strategy was drafted and subsequently refined following two rounds of team discussion. The search terms were related to doctors, PA, promotion and perceptions (Table 1), and the final search strategy was adapted for each database and logged. The Boolean operator ‘AND’ was used between categories and ‘OR’ within categories.

**Table 1:** Search terms used for systematic searching

Target population	Physical activity	Perceptions	Promotion
doctor*, physician*, surgeon*, "general practitioner*”, gp*, "healthcare profession*", "health care profession*", “primary care”	“physical activit*”, exercis*, activit*, walk*, green*	perception*, view*, perspective*, thought*, think*, opinion*, attitude*, believe*, belief*, idea*, barrier*, influenc*, opportunit*, qualitative	prescription*, prescrib*, promot*, counsel*, refer*

The asterisk symbol (*) indicates truncation which is used to instruct online databases to search for alternative word endings

Electronic literature databases were searched from 2011 on the basis of the publication of the Chief Medical Officer PA recommendations [14]. Although these were updated in 2019, the 2011 aerobic recommendation for accumulating 150 min of moderate intensity PA per week is still in use. The final search was carried out on 9 October 2022 and an inclusive approach to inclusion and exclusion criteria was taken (Table 2). Studies were excluded if they did not explore the perceptions of doctors. However, if a study explored the perceptions of other HCPs as well as doctors, then this was included, but only data relating to doctors were extracted. Final search terms are available in Additional file 1.

**Table 2:** Inclusion and exclusion criteria

Inclusion criteria	Exclusion criteria
➾ Full-text articles published in peer-reviewed journals and grey literature ➾ Articles published in English ➾ UK-based studies ➾ Any healthcare setting (e.g., general practice or hospital-based) ➾ Any kind of doctor (e.g., general practitioners, surgeons, physicians, junior doctors) ➾ Any kind of PA promotion (e.g., promotion, referral, prescription, social prescribing) ➾ Real or simulated patients	➾ Abstracts without full-text available ➾ Articles investigating only other healthcare professionals (e.g., nurses, physiotherapists, pharmacists) or medical students

### Stage 3: Study selection

Identified studies were uploaded to Covidence™ software, which automatically removed duplicates. All 100% of records at the title and abstract stage, and subsequently 100% of records at the full-text stage, were independently screened by two members of the author team. Conflicts were discussed and resolved by team discussion, and criteria were modified if required, for example ordering the exclusion criteria.

### Stage 4: Charting the data

The data extraction table was drafted by GW using Microsoft Excel and was refined following team discussion. Data were independently extracted (100%) by two members, with any discrepancies and uncertainties discussed and resolved through team discussion. Data points extracted relating to demographics included:

- Authors
- Title
- Location (UK-wide, Scotland, England, Wales, Northern Ireland)—if studies included data from both Ireland and Northern Ireland, only data that reflected the views of doctors in Northern Ireland were extracted
- Setting (e.g., primary care/community care, secondary care, mixed primary/secondary care)
- Type of doctor (e.g., GP, mixed doctor group, consultant physicians, junior doctors)
- Type of patient (e.g., unspecified, adults with specified medical conditions, children/young people with specified medical conditions)

Other data points collected included study methods (e.g., qualitative, quantitative, mixed-methods) and any theoretical models used if they were relevant (e.g., theory of planned behaviour, theoretical domains framework). Details of study aims and limitations as noted by the study authors or as interpreted by the authors of this review were extracted. The key findings extracted reflected the factors that influenced PA promotion by doctors. In studies that presented qualitative data, all relevant statements were extracted verbatim. In studies that reported quantitative data, key concepts in statements or survey items were extracted in addition to quantitative data.

The final data extraction table is available in Additional file 2. Quality assessment of individual papers was not undertaken, as it is not a recommended element of scoping reviews [28].

### Stage 5: Collating, summarising and reporting

Study characteristics were reported using descriptive numerical analysis, displaying frequencies of demographics and the type of study (qualitative, quantitative or mixed-methods). Influencing factors for PA promotion were reported using a narrative summary and following a qualitative thematic analysis of the data. We conducted a ‘codebook’ version of thematic analysis [31], where we adopted a qualitative philosophical approach to this aspect of analysis whilst following a structured coding approach and with our main themes conceptualised as domain summaries, and named accordingly to reflect factors previously identified in the literature (e.g., knowledge and training). Consistent with codebook thematic analysis, measures to enhance validity of the findings or quality of the process, such as inter-rater reliability or coder consensus, were not conducted [31]. Additionally, given the review design, we could not conduct processes that may be applicable in primary qualitative studies such as member reflections [32]. Therefore, our attempts to enhance rigour focussed on the use of critical friends—members of the research team who provided critical discussion and challenged the codes and themes developed by the lead author [32]. Finally, gaps in the research were mapped using a table, organised by study setting, type of doctor, type of PA promotion, study location and patient group.

## Results

### What research exists examining the perceptions of UK doctors on PA promotion?

**Fig. 1:**
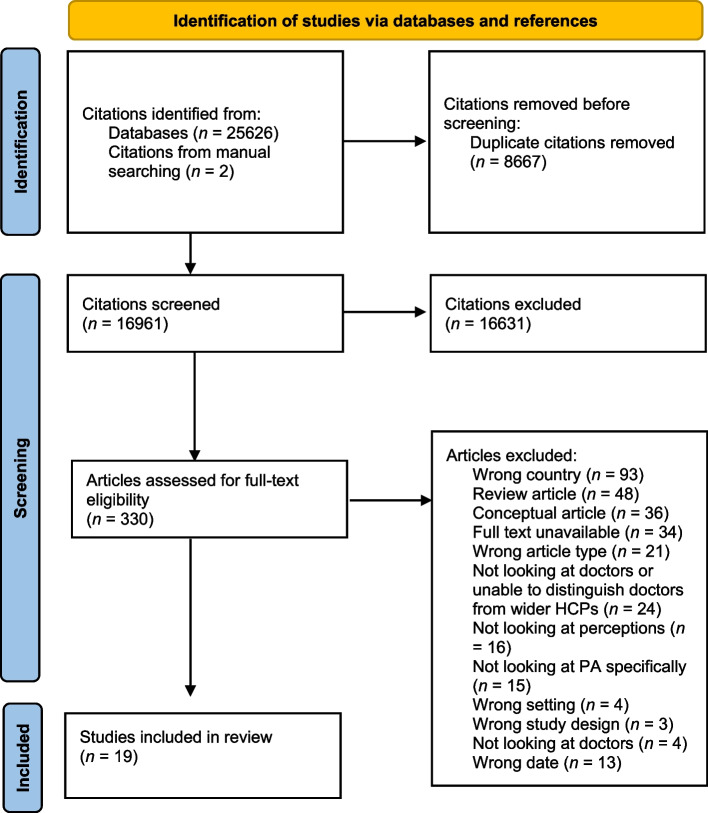
Study selection flowchart

In total, 25,628 references were identified for screening (25,626 from database searching and 2 from manual searches) and a total of 330 studies were included for full-text screening. There was a 4.9% disagreement rate at full-text screening. In total, 19 studies were included in final analysis. A study selection flowchart is presented in Fig. 1.

Of the 19 studies meeting the inclusion criteria, most used qualitative methods (63%, *n* = 12) and most were set in primary care/community settings (68%, *n* = 13) involving GPs. Only 32% (*n* = 6) of studies used theoretical models as frameworks to explore the perceptions of doctors and these models varied (see Table 3 for full-study characteristics).

**Table 3:** Study characteristics

**Study feature**	**Number of studies (%)**^a^
**Study methods**	
Qualitative (semi-structured interviews/questionnaires)	12 (63)
Quantitative (questionnaires)	3 (16)
Mixed-methods	4 (21)
**Location**	
UK-wide	8 (42)
England	8 (42)
Scotland	1 (5)
Wales	1 (5)
Northern Ireland and Ireland (only data from Northern Ireland extracted)	1 (5)
**Setting**	
Primary care/community	13 (68)
Secondary care	3 (16)
Mixed primary/secondary care	2 (11)
Cancer care	1 (5)
**Type of doctor**	
GP	12 (63)
Mixed-doctor group	3 (16)
Consultant physicians	3 (16)
Junior doctors	1 (5)
**Sample size: number of doctors**	
0–10	7 (37)
11–25	5 (26)
26–100	3 (16)
101–500	2 (11)
501 +	2 (11)
**Patient type**	
Unspecified patient group	7 (37)
Adults with specified medical conditions (e.g., chronic knee pain, non-alcoholic fatty liver disease, adults)	7 (37)
Children/young people with specified medical conditions (e.g., diabetes)	3 (16)
Preconception females	1 (5)
Older adults	1 (5)
**Theoretical model adopted**	
No model identified	15 (79)
Theory of planned behaviour	2 (11)
Theoretical domains framework	1 (5)
Social cognitive theory	1 (5)

^a^ Percentages may not add up to 100% due to rounding

### What factors influence PA promotion by UK doctors?

#### Knowledge and training

Lack of knowledge was frequently identified as a barrier to promoting PA by UK doctors. Evidence from qualitative research [33–39], mixed methods research [19, 40–42] and quantitative research [43–45] found that doctors reported insufficient knowledge in PA guidelines and benefits [19, 33, 34, 40, 43–45], effectiveness of PA promotion/exercise referrals (ERs) [35, 36, 39, 41], how to promote PA [43, 44], the referral process for exercise referral (ER) schemes and sport and exercise medicine (SEM) clinics [37, 39, 41, 42] and patient safety liability in exercise programmes [35]. In their mixed method study of 15 GPs delivering PA advice to patients with diabetes, Kime et al. found that no doctors were familiar with guidelines relating to PA and diabetes [40], and qualitative research involving four consultants working with children with type 1 diabetes found that some doctors questioned the effectiveness of the guidelines they were implementing [36]. A qualitative study of GPs using ER schemes for patients with depression found that information regarding the schemes are frequently absent or out of date, the referral process is sometimes unclear and limited feedback means efficacy is difficult to assess, although what feedback is received is positive [39]. In a qualitative study of 11 junior doctors, some felt the essence of medical training is illness diagnosis and disease management, with one participant expressing that ‘we are more scared of missing an ill patient rather than preventing an illness a patient does not have from happening in the future’ [33].

Most studies found that when doctors felt they had good knowledge of PA benefits, guidelines and screening tools, they were more encouraged to participate in PA promotion/ERs [19, 34, 38, 43, 44, 46]. In their study of 1013 GPs, Chatterjee et al. found that almost twice as many GPs who were unfamiliar with relevant guidelines reported being less confident raising PA with patients than those who were familiar with guidelines [19], and increased exercise promotion was found among 835 GPs that had read the relevant NICE guideline for managing their patients with osteoarthritis [43]. However, in their qualitative study of 166 fully qualified GPs and 65 GP registrars, Wheeler et al. found that although most fully qualified GPs felt sufficiently knowledgeable to advise inactive patients, they did not always provide PA counselling, and fewer still would always refer or signpost inactive patients to exercise programmes or local PA opportunities [44]. In a qualitative study of 15 GPs participating in a randomised control trial of facilitated PA and usual care for depression, the extent to which GPs promoted PA was variable, despite their involvement in the trial. This variation did not appear to be related to their awareness of an evidence base [47]: only one GP referred to NICE guidance to support recommending PA to patients, but others stated often anecdotal evidence of the benefits of PA for other patients to improve mood. Three of the 15 GPs interviewed felt there was insufficient evidence to recommend PA to depressed patients [47]. In a qualitative study of challenges and approaches to green social prescribing, one GP described the benefits to regular outdoor exercise for chronic pain patients, but felt that some patients did not want PA as a drug-free alternative management for their chronic pain [46]. Although insufficient training was identified as a barrier [33, 40, 45, 48] and provision of education identified as a facilitator [33, 40, 41] to PA promotion, it was suggested that unless doctors had a particular interest in or enthusiasm for PA, it was unlikely they would seek further training [40].

#### Personal interest and activity

Qualitative, mixed method and quantitative research suggest a personal interest in the benefits of PA motivates doctors to promote it [35, 36, 38, 40, 41, 43, 46, 47]. Some doctors view themselves as role models. Qualitative studies of 10 and 31 GPs, conducted by Cunningham et al. and Din et al., respectively, found that GPs felt their personal PA levels make them a credible source of advice for patients [35, 38]. A qualitative study of six GPs found that although all GPs interviewed were positive about social prescribing, those who participated themselves were more likely to recommend a particular activity to patients; for example, one GP in this study had been involved in Parkrun for many years and routinely recommended it to both patients and staff [46]. In contrast, a mixed method study of GPs indicated that a lack of personal PA/interest in PA promotion acted as a barrier [41]. Qualitative and mixed method studies of GPs found that if they are themselves inactive or overweight, they may feel uncomfortable/hypocritical advising patients to exercise [35, 41]. Indeed, in their qualitative study of 11 junior doctors, Osinaike and Hartley found that the doctors felt the success of their PA advice may be influenced by patients’ perceptions of them as a role model [33]. This is supported by a qualitative study of 15 GPs using facilitated PA for treatment of depression which found that some GPs felt it was important to discuss their own views to validate the efficacy of PA as a non-drug treatment for depression [47].

#### Time

Most studies, regardless of design, identified insufficient time as a prominent barrier to PA promotion/ER use [33–35, 38–41, 43–45, 49] with the perception that doctors should prioritise other issues within consultations [34–36, 40, 41]. Insufficient time was identified in both primary and secondary care settings: in their study of 231 GPs and GP registrars, Wheeler et al. reported that 91% of fully qualified GPs perceived that insufficient time limits them discussing PA [44]. Although both junior doctors [33] and consultants [45] identified time constraints as a barrier in hospital settings, in a qualitative study of 11 junior doctors it was suggested that lack of time was not such a major hindrance to junior doctors, and since they are sometimes allocated more time with patients than their senior colleagues, they may be in a more favourable position to undertake PA counselling [33].

#### Resources

Qualitative and mixed-method evidence indicates that when doctors felt they had suitable resources, including tools [48, 50] and e-referrals/simple referrals [41], they felt more able to promote PA. In their mixed-method study employing an online survey of 56 GPs and interviews with seven GPs Buckley et al. found that incentivising PA promotion may encourage more doctors to engage in PA promotion [41]. A qualitative study of 15 GPs in a randomised control trial comparing usual care for depression with usual care plus facilitated PA found that doctors felt that facilitated PA needs to be cost-effective and well-resourced to be a viable treatment option, and evidence of this cost-effectiveness is needed [47]. Insufficient financial resources were identified as a barrier in a qualitative study of 31 GPs [35] in which participants suggested money could be used for management of other serious health conditions rather than promoting PA. One GP in a qualitative study felt that there was an ongoing lack of financial support for management options including ER schemes that do not provide a ‘quick fix’ [39]. Quantitative studies found that other, unspecified insufficient resources [44, 45] act as barriers to doctors engaging in PA promotion. Furthermore, insufficient incentives were identified as a barrier in Wheeler et al.’s study of 231 GPs and GP registrars [44]. A lack of knowledge-based resources were identified in a mixed-method study of GPs [41], and a lack of clinical mentorship/supervision was identified as a barrier to PA promotion in Osinaike and Hartley’s qualitative study of 11 junior doctors [33].

#### Confidence

Qualitative and mixed-method studies showed that some doctors feel confident promoting PA [33, 42, 44]. In their mixed-methods study of 244 GPs, Kassam et al. found that 82% felt confident giving less active patients PA advice [42]. However, other qualitative and mixed-method studies identified a lack of confidence hindered PA promotion/ER for some doctors [33, 34, 37, 40]. Interviews with 15 GPs conducted by Kime et al. investigated how prepared GPs felt delivering PA advice to patients with diabetes, and found that some GPs felt out of their depth giving PA advice in general, and particularly concerning PA and diabetes [40]. In their qualitative study of eight GPs in Scotland, Sissons et al. found that some lacked confidence in the effectiveness of their advice and felt their guidance on PA for preconception women may be less effective than other information sources [34]. In a study of 33 doctors with haematological cancer patients, approximately two thirds of the medical professionals felt that they lacked confidence giving PA advice to patients during cancer treatment, and less than half felt confident giving PA recommendations after treatment [37]. In this same study, a fifth of doctors agreed with a statement that they knew where or to whom to refer patients who needed support to be more active during haematological cancer treatment. A quantitative study of 231 doctors by Wheeler et al. found that 62% of fully qualified GPs felt very/extremely confident giving general PA advice to patients, compared with 34.6% of GP registrars. However, this study also found that over 26% of fully qualified GPs and 65% of GP registrars felt not very/not at all confident in giving general PA advice to their patients [44]. In their qualitative study of 11 junior doctors, Osinaike and Hartley found that observing good examples of PA counselling can help junior doctors feel ‘empowered’ [33].

#### Perceived role

Doctors, based in both primary and secondary care, generally believed they should play a role in PA promotion/counselling [33, 34, 43, 44], with one participant in a study of junior doctors going so far as to say ‘I think I am letting my patient down for not talking to them about PA’ [33]. A qualitative study of 15 GPs suggested that patients would be more likely to adhere to PA if it was recommended by a GP [47]. However, it appears that both GPs [34, 35, 41] and hospital-based doctors [33] feel that they may not be best suited to provide this. In interviews with seven GPs, Buckley et al. found that they felt there was an overreliance on them, ‘work is always being dumped on the GP’ [41]; PA is not their sole responsibility and other HCPs may be better positioned and have more time to provide PA advice [41]. A qualitative study of 11 junior doctors found that some based in hospital felt that primary care was a more appropriate setting for disease prevention interventions than hospital, although others felt PA counselling could still be undertaken in hospitals [33]. Several qualitative and mixed-method studies demonstrated that doctors felt that other HCPs, such as practice nurses, were better placed to provide PA advice [33, 34, 41]. There was no consensus on whose role PA promotion is and how doctors should be involved [36, 49], with some doctors believing patient self-management may have greater potential for increasing patient PA [34, 35].

#### Patient relevance

Some doctors feel PA promotion is not relevant for every patient [19, 35, 41, 44, 45, 47] or reported discussing it only if patients raised it during consultations [34, 37]. In their study of 244 GPs, Kassam et al. found that doctors are more likely to refer young patients with musculoskeletal problems or elite athletes to SEM clinics than patients with chronic conditions who are interested in starting to exercise [42]. A qualitative study of GPs using ERs for the management of depression found that GPs perceived some patient-related barriers as obstacles to referral, including how patients with depression may struggle to motivate themselves to start PA and sustain this without supervision, be averse to group activities or struggle with the financial aspects of PA (the latter being related to when discounted exercise classes ended in this study) [39]. In a qualitative study involving only one consultant, Shelly et al. found that this doctor felt reluctant to inform patients of their inactivity, fearing this may have a detrimental rather than an encouraging effect [50]. This was echoed by a GP interviewed in a qualitative study of 15 GPs using facilitated PA as part of treatment for depression [47] and by a GP in a qualitative study investigating the use of ERs in management of depression, who felt counselling to facilitate initial motivation to exercise had ‘made things worse’ [39].

### What are the gaps in the research regarding perceptions of UK doctors on PA promotion?

Using the data from the 19 studies included in this scoping review, the degree of evidence provided by these articles has been categorised based on the level of existing evidence, as previously utilised by Williamson et al. [51]. Evidence was considered strong when three or more studies existed; minimal, when one–two studies existed; and non-existent, when zero studies existed (Table 4). This helped to highlight the areas where there are gaps in the literature. Studies more frequently focused on the perceptions of GPs in a primary care setting rather than doctors working in secondary care, and most did not target a specific age group. Gaps exist in the literature exploring doctors in primary care looking after children and there are few region-specific studies.

**Table 4.**
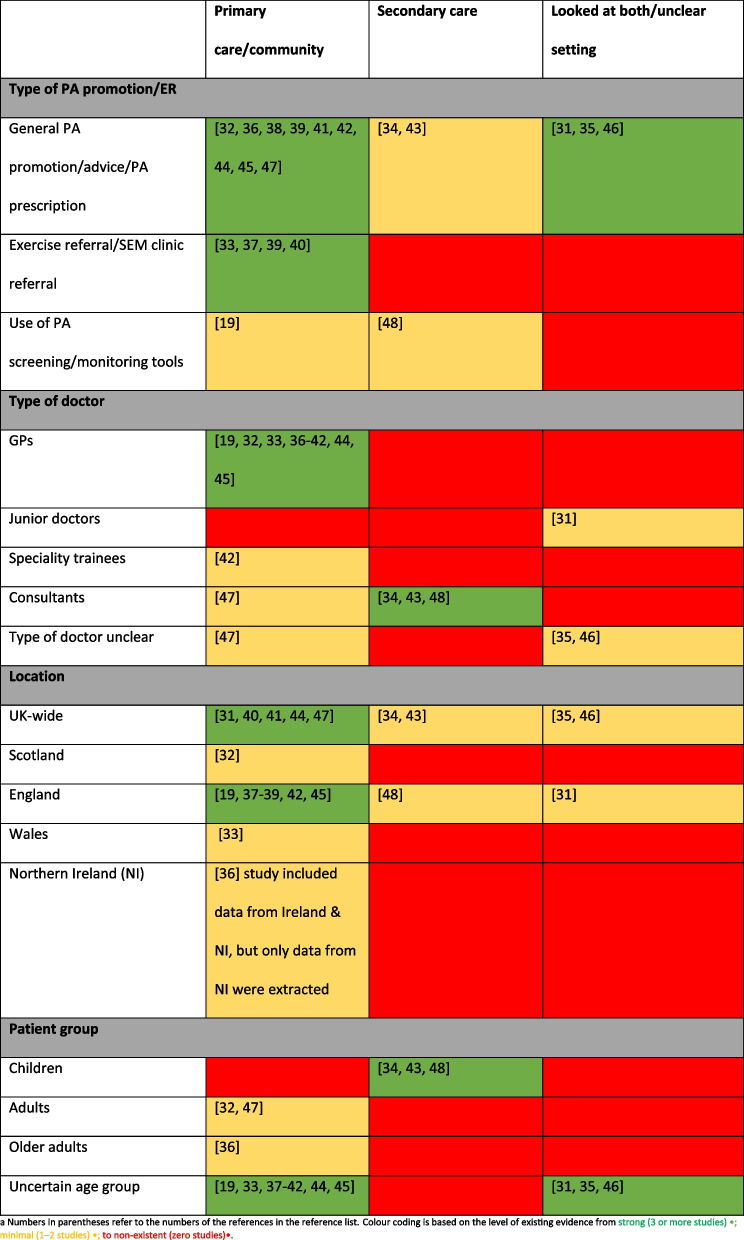
Spread of included studies

## Discussion

### Summary of principal findings

**Fig. 2:**
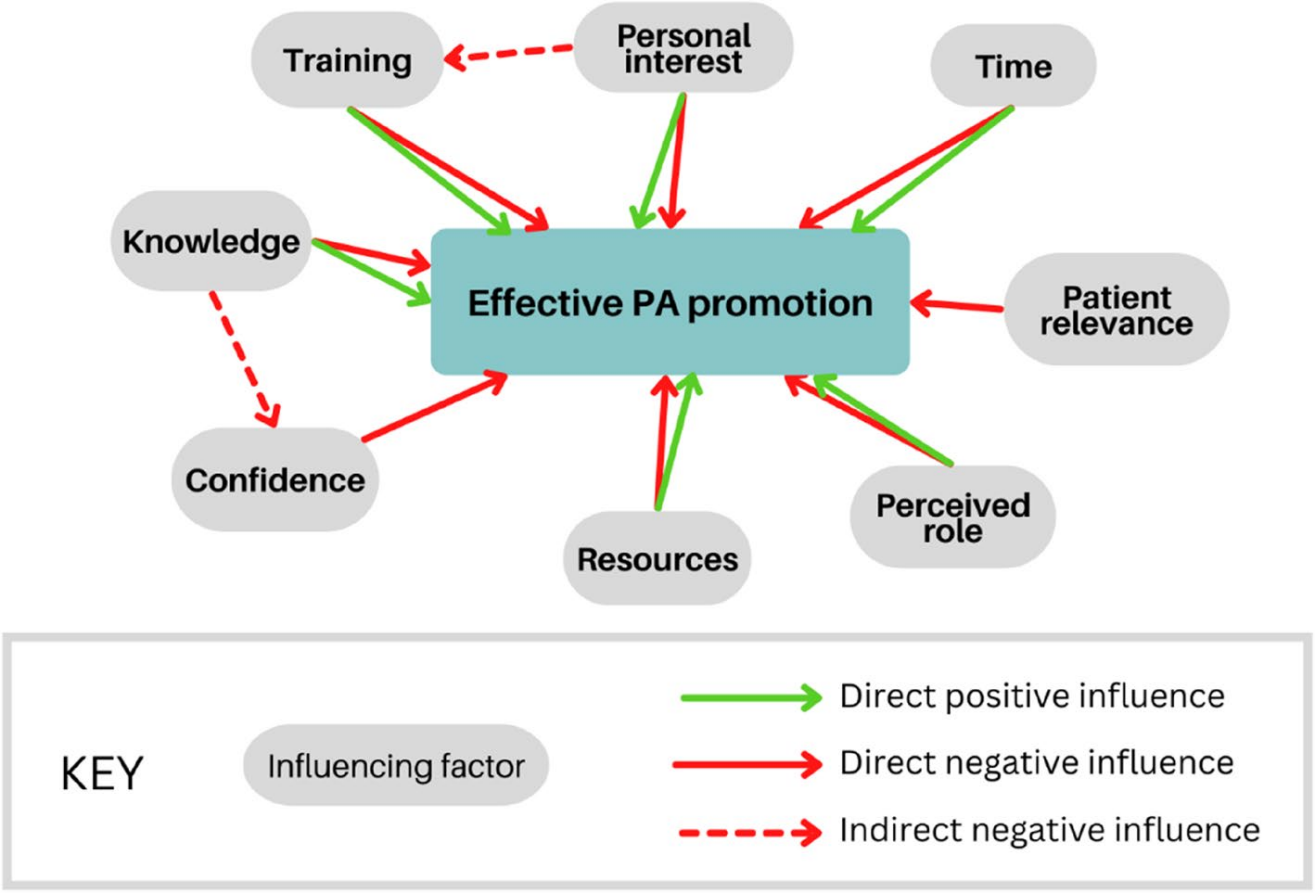
A conceptual map of factors influencing PA promotion by doctors in the UK

PA promotion by doctors is, by nature, complex and influenced by cognitive, behavioural and environmental factors. To our knowledge, this is the first study to synthesise research specifically examining perceptions of PA promotion from doctors in the UK. Despite a comprehensive search strategy, the depth of research was limited, averaging approximately only two studies published per year in the last decade. Of the data published, most explore the perceptions of UK GPs working in primary care. Several themes were identified as key influencing factors experienced by UK doctors: knowledge and training, personal interest, time, resources, confidence, belief in personal role and perceived relevance to the patient. These factors do not operate in isolation and can be bi-directional, acting as barriers and/or facilitators within an interlinked network (Fig. 2). For example, the factor of knowledge overlaps with the factor of confidence, in that doctors receiving training or seeing examples of good practice would resultingly have increased confidence and more likely to refer patients to programmes or prescribe exercise prescriptions of some nature.

These findings, taken from literature published since 2011, provide evidence that doctors continue to experience multiple barriers in promoting PA to their patients. The themes identified in this study can be used as a base for developing potential intervention strategies to support the promotion of PA by doctors.

### Comparison with existing literature

The principal findings here corroborate previous reviews investigating global perceptions of HCPs [18, 24, 25, 27], suggesting that efforts to overcome these previously identified barriers to PA promotion have had limited success.

This contemporary review indicates that UK doctors believe they should play a role in PA promotion, concurring with older UK-based works [52, 53]. Similarly, a global review found that most primary care providers viewed PA counselling as important and that they should be involved [27]. However, this review identified that some doctors feel they are not in the best position to promote it, supporting the notion that HCPs still do not unanimously agree on who should be involved and how [18, 27]. The clinical setting that doctors were working in influenced their perception of who was best positioned to undertake disease prevention interventions and, although many GPs felt it was part of their role, some felt that there was an overreliance on them for lifestyle interventions [41]. It is plausible that these findings support previously noted misconceptions that PA is only a preventative measure [54] and not an effective method of treatment. Another theme identified in this review was that there appears to be a lack of support for therapies such as PA that do not provide a ‘quick fix’, i.e., an immediate benefit is not always observed [39], possibly in part because of the perception of patients that the role of doctors is to prescribe medications [46]. It has been suggested that more understanding of when patients are most receptive to PA interventions in primary care is required [25], and this finding further demonstrates this need. Although junior doctors feel they should play a role in PA promotion [33], this review found limited data exploring the views into the role of other hospital-based doctors of this PA promotion role. Thus, further data are required to comment on whether or not there is a difference in the perceived role between the perceptions of primary and secondary care doctors.

Previous research indicated that insufficient confidence, knowledge and training hindered doctors from engaging with PA promotion [18, 24, 27]. Despite the recent provision of easily accessible recommendations and resources for doctors to use, including NICE guidelines [6], the FSEM’s ‘Moving Medicine’ resource [16] and recommendations from the Royal College of Physicians [15], this review provides evidence that these barriers remain prevalent. This review found that when doctors had knowledge of the relevant guidelines, they had increased confidence [19] and more readily promoted PA to their patients [43]. It would, therefore, be useful to determine how often doctors access such resources, as it is reasonable to suggest that increasing exposure to these tools may enable doctors to engage. Unexpectedly, Searle et al. found that the extent to which GPs promoted PA did not appear to be related to their awareness of an evidence base, although GPs cited other reasons for why they felt PA may be useful in the treatment of depression [47]. However, it should be noted that this finding was not replicated by other studies included in this review.

The role of PA training within medical school curricula remains a potentially influential factor on PA promotion. This review found some evidence of doctors receiving no PA training during medical school [33] and identified the perception that some doctors believe medical training is based around diagnosis and disease management of sick patients rather than the prevention of disease in the future. Previous work concluded that UK medical students lacked confidence prescribing PA to patients [55], and it was previously suggested that without inclusion of PA questions in medical school examinations, it (and wider lifestyle medicine) was unlikely to be considered a core competency by students [55]. Data in this review suggested that unless doctors were particularly interested in PA, some believed it was unlikely they would seek additional training in the area, but that when doctors have better knowledge, they are more likely to engage with PA promotion. Similarly, observing good examples of PA counselling may help doctors feel ‘empowered’ [33]. Therefore, making PA training a mandatory and examined component of medical training, as well as clarifying guidance about how to incorporate PA counselling and other communication types into consultations and providing opportunities to observe good examples of this, may enable more effective PA promotion by doctors.

Findings from this review suggest that even for fully qualified GPs with sufficient knowledge, physical inactivity as a health behaviour was less likely to be discussed than other modifiable health behaviours such as smoking, and the delivery of PA counselling or referral to programmes and opportunities was not always conducted [44]. Whilst the specific reasons for this remain unclear, there is some evidence that doctors can be uncertain about the effectiveness of ER schemes [35, 39] with a need for provision of clear information about these schemes, including scheme content, eligibility and patient progress [41]. These findings echo a review published in 2022, in which the authors highlight that evidence is required to show doctors that behavioural interventions have an important place in patient-centred and evidence-based medical practice [25]. Although exercise is advocated within clinical care services, comparison of clinical exercise provision in the UK found that staff roles and qualifications across services were inconsistent [56]. It was previously suggested that greater regulation is required to evaluate and compare services and that standardising staff roles and the qualifications required may provide reassurance to referring doctors and patients that these services are effective and safe [56], thereby increasing the confidence in and subsequent use of these services.

Consistent with previous reviews [18, 24, 27], this review highlighted insufficient time as a pertinent barrier to PA promotion by doctors and HCPs. A novel finding was that junior doctors appeared to experience this (time as a barrier) to a lesser degree than their senior colleagues [33]. In addition, the inclusion of brief PA advice on hospital discharge summaries was identified as a possible way to ensure the topic is explored with the patient [33], and since these are often completed by junior doctors, this may offer an intervention strategy. Furthermore, involvement of the wider HCP team may assist in tackling the time barrier; indeed, in their review, Hébert et al. [27] found that primary care physicians struggled more with time than their nursing colleagues. Delegating some counselling responsibility to this professional group would potentially alleviate some pressure on doctors, while ensuring it remains a priority within primary care. In fact, older research suggested that PA interventions delivered by allied health professionals (health educators and physiologists), or by allied health professionals in conjunction with physicians, produced the best long-term results in patients [57]. Countries including Canada, Australia and the USA utilise clinical exercise physiologists, recognising their expertise in supporting PA engagement [56, 58, 59], but this is not currently the case in the UK, where there is no recognition or regulation of the profession [56] despite growing calls for this to try and increase PA in patients. ‘Moving Medicine’ [16] provides resources for clinicians to tailor PA counselling according to the time they have available—using as little as one minute—and provides a structure to follow. Increasing awareness of tools such as these would enable clinicians to use even small amounts of time for effective PA promotion.

Our review provides evidence that doctors still do not believe PA promotion is relevant for all patients, and that a personal interest in the benefits of PA motivates doctors to promote it, corroborating older evidence [18, 24]. This questions the suitability of current training for doctors because UK guidelines recommend that all adults should aim to achieve the guidelines [14]. Previous work also suggested physically active doctors were more likely to encourage patients to be active than their inactive colleagues [24, 27, 60–62], and findings here concur, demonstrating some inactive/overweight doctors feel uncomfortable advising patients. Physical inactivity is prevalent even within the medical community [63], and these findings suggest that addressing this and increasing doctors enthusiasm in PA could translate into more doctors engaging in counselling with patients. Global evidence investigating HCPs identifies insufficient resources as a key barrier to PA promotion [18, 27]. Hébert et al. identified environmental context and resources as a key barrier to PA promotion by HCPs [27], but insufficient resources were identified less frequently in this review. It is possible that this reflects the global and broad nature of those previous reviews, where available resources may vary. However, this has still been identified as an area for improvement to enhance PA promotion in the UK.

### Literature gaps and further research

This review demonstrates the existence of evidence examining PA promotion by UK doctors, but highlights an imbalance within the breadth of research, with most studies investigating the perceptions of GPs in primary care. This may well reflect that general practice is often considered the optimal location for health promotion but obtaining more information regarding the perceptions of hospital-based doctors, and doctors managing specific patient groups are warranted. This would enable further insight and comparison between primary and secondary care, examining if one setting and group of professionals may be better suited to influence PA than another.

A standardised approach, using first a set of general, followed by specific questions that can be asked of doctors of all types in all settings across the UK may be useful to develop a comprehensive understanding of the factors influencing whether doctors promote PA or not. The COM-B model from within the Behaviour Change Wheel offers one viable approach for researchers to investigate this topic [64]. It would ensure data are aligned with a particular source of behaviour and would permit comparisons between groups in subsequent evidence syntheses.

Further, linking with intervention functions within the Behaviour Change Wheel would permit the investigation of what is required to overcome identified barriers and aid intervention development. The findings from this review suggest that a multi-component intervention, involving several functions is required to overcome the barriers identified. As an example, the lack of confidence and knowledge identified in this review indicates that psychological capability is a key source of behaviour and that although guidelines exist, these, along with other available resources, need to be communicated more effectively to doctors through the use of an intervention incorporating training and education [64]. Wider policy support, to ensure such an intervention is made mandatory within medical training, may lead to changes in practice that could be maintained. Additionally, this review provides evidence that enablement would be a relevant function within such an intervention, given the complex, interrelated set of problems or barriers faced by doctors [64]. Building on the findings that doctors view themselves as a credible source, and that seeing examples of best practice promotion would be advantageous, techniques to promote the function of modelling would appear pertinent [64].

### Strengths and limitations

This review aimed to provide an insight into the available evidence on the perceptions of factors influencing PA promotion by the UK doctors. Key strengths were the comprehensive search strategy and inclusion of varied study designs, enabling a thorough overview of published evidence. Further strengths include the use of an established protocol and including different forms of intervention, from simple PA advice to exercise referral schemes, in our broad definition of PA promotion. Including studies primarily investigating perceptions of wider HCPs but extracting only data about doctors allowed the inclusion of data that would otherwise have been excluded from the search. The frameworks adopted by this review advocate for the use of consultations with stakeholders to assist in identifying research questions [28]; however, time constraints associated with the project meant this was not possible. The nature of scoping reviews means that the evidence included was not quality appraised: many included studies had small sample sizes, particularly those investigating the wider HCP team, and in one study, only one doctor’s views were explored [50]. Therefore, further research may be required to investigate and corroborate or disregard some of the themes and issues discussed here. Despite undertaking an extensive database search, only 19 studies were included. Although a relatively small number, these 19 studies published in the last decade best reflect current perceptions and provide insight into factors influencing PA promotion within the timeframe of current PA guidelines. This small evidence base meant it was not possible to comment on which doctors are best able to influence PA, although it should be noted it was not a stated purpose of this scoping review to do so.

## Conclusions

Our findings highlight that multiple barriers still exist which prevent UK doctors’ from engaging with PA promotion with their patients. Knowledge and training, personal interest, time, resources, confidence, belief in personal role and perceived relevance to the patient were identified as key influencing factors experienced by doctors. These influencing factors concur with older research, suggesting that previous efforts to overcome them have had limited success and that further intervention to address these factors is required. In light of these persistent barriers, several recommendations have been made to inform the development of future interventions to address these barriers and support the delivery of PA promotion by doctors.

## Supplementary Information

**Additional file 1: Table S1.** Final search terms.

**Additional file 2: Table S2.** The final data extraction table.

## Data Availability

The data supporting the results in this article can be found in previously published manuscripts. The ‘dataset’ (i.e., fully extracted study information) analysed during this study is available on reasonable request.

## References

[1] Warburton D, Bredin S (2017). Health benefits of physical activity: a systematic review of current systematic reviews. Curr Opin Cardiol.

[2] Zubala A, MacGillivray S, Frost H, Kroll T, Skelton DA, Gavine A (2017). Promotion of physical activity interventions for community dwelling older adults: a systematic review of reviews. PLoS ONE.

[3] NHS. Statistics on obesity, physical activity and diet, England, 2020. 2020. Available from: https://digital.nhs.uk/data-and-information/publications/statistical/statistics-on-obesity-physical-activity-and-diet/england-2020. Accessed 09/10/2021.

[4] Scottish Government National Statistics. The Scottish health survey; 2019. Available from: https://www.gov.scot/binaries/content/documents/govscot/publications/statistics/2020/09/scottish-health-survey-2019-volume-1-main-report/documents/scottish-health-survey-2019-edition-volume-1-main-report/scottish-health-survey-2019-edition-volume-1-main-report/govscot%3Adocument/scottish-health-survey-2019-edition-volume-1-main-report.pdf. (Accessed 19/11/2022).

[5] Welsh Government. Adult survey (national survey for Wales): April 2021 to March 2022; 2022. Available from: https://gov.wales/adult-lifestyle-national-survey-wales-april-2021-march-2022. (Accessed 19/11/2022).

[6] National Institute for Health and Clinical Excellence. Physical activity: brief advice for adults in primary care PH44. 2013. Available from: https://www.nice.org.uk/guidance/ph44/resources/physical-activity-brief-advice-for-adults-in-primary-care-pdf-1996357939909. (Accessed 19/11/2022).

[7] Public Health England. Physical activity: applying All Our Health. 2019. Available from: https://www.gov.uk/government/publications/physical-activity-applying-all-our-health/physical-activity-applying-all-our-health. (Accessed 19/11/2022).

[8] Bauman A, Reis R, Sallis J, Wells J, Loos R, Martin B (2012). Correlates of physical activity: why are some people physically active and others not?. Lancet.

[9] World Health Organization. Global action plan on physical activity 2018–2030: more active people for a healthier world. World Health Organization; 2018. Available from: https://apps.who.int/iris/bitstream/handle/10665/272722/9789241514187-eng.pdf?sequence=1&isAllowed=y. (Accessed 19/11/2022).

[10] Royal College General Practitioners. Clinical Priorities. 2019. Available from: https://www.rcgp.org.uk/clinical-and-research/our-programmes/clinical-priorities.aspx. (Accessed 09/10/2021).

[11] Sallis R (2011). Developing healthcare systems to support exercise: exercise as the fifth vital sign. Br J Sports Med.

[12] Information Service Division Scotland. Practice Team Information (PTI) Annual Update (2012/13). Available from: https://www.isdscotland.org/Health-Topics/General-Practice/Publications/2013-10-29/2013-10-29-PTI-Summary.pdf?3695315123. (Accessed 19/11/2022).

[13] Orrow G, Kinmonth A, Sanderson S, Sutton S (2012). Effectiveness of physical activity promotion based in primary care: systematic review and meta-analysis of randomised controlled trials. BMJ.

[14] Department of Health and Social Care. UK chief medical officers’ physical activity guidelines. DH, London; 2019. Available from: https://assets.publishing.service.gov.uk/government/uploads/system/uploads/attachment_data/file/832868/uk-chief-medical-officers-physical-activity-guidelines.pdf. (Accessed 19/11/2022).

[15] Royal College of Physicians. Exercise for life. Physical activity in health and disease. Recommendations of the sport and exercise medicine committee working party of the royal college of physicians; 2012. Available from: https://www.rcplondon.ac.uk/file/1287/download. (Accessed 19/11/2022).

[16] Faculty of Sport and Exercise Medicine UK. Moving Medicine. 2022. Available from: https://movingmedicine.ac.uk/. (Accessed 10/04/2022).

[17] Cantwell M, Walsh D, Furlong B, Moyna N, McCaffrey N, Boran L, et al. Healthcare professionals’ knowledge and practice of physical activity promotion in cancer care: challenges and solutions. Eur J Cancer Care (Engl). 2018;27(2): e12795. 10.1111/ecc.12795 29193416

[18] Albert F, Crowe M, Malau-Aduli A, Malau-Aduli B (2020). Physical activity promotion: a systematic review of the perceptions of healthcare professionals. Int J Environ Res Public Health.

[19] Chatterjee R, Chapman T, Brannan M, Varney J (2017). GPs’ knowledge, use, and confidence in national physical activity and health guidelines and tools: a questionnaire-based survey of general practice in England. Br J Gen Pract.

[20] O’Brien S, Prihodova L, Heffron M, Wright P. Physical activity counselling in Ireland: a survey of doctors’ knowledge, attitudes and self-reported practice. BMJ Open Sport Exerc Med. 2019;5(1): e000572. 10.1136/bmjsem-2019-000572 PMC6677951 31423324

[21] Patel A, Schofield G, Kolt G, Keogh J. General practitioners’ views and experiences of counselling for physical activity through the New Zealand green prescription program. BMC Fam Pract. 2011;12:119. 10.1186/1471-2296-12-119 PMC3233500 22044577

[22] Swinburn B, Walter L, Arroll B, Tilyard M, Russell D (1997). Green prescriptions: attitudes and perceptions of general practitioners towards prescribing exercise. Br J Gen Pract.

[23] Bull F, Schipper E, Jamrozik K, Blanksby B (1995). Beliefs and behaviour of general practitioners regarding promotion of physical activity. Aust J Public Health.

[24] Huijg J, Gebhardt W, Verheijden M, van der Zouwe N, de Vries J, Middelkoop B (2015). Factors influencing primary health care professionals’ physical activity promotion behaviors: a systematic review. Int J Behav Med.

[25] Hall L, Thorneloe R, Rodriguez-Lopez R, Grice A, Thorat M, Bradbury K, et al. Delivering brief physical activity interventions in primary care: a systematic review. Br J Gen Pract. 2022;72(716):209–16. 10.3399/BJGP.2021.0312 PMC8597771 34782318

[26] Ribera A, McKenna J, Riddoch C (2006). Physical activity promotion in general practices of Barcelona: a case study. Health Educ Res.

[27] Hébert E, Caughy M, Shuval K (2012). Primary care providers’ perceptions of physical activity counselling in a clinical setting: a systematic review. Br J Sports Med.

[28] Arksey H, O’Malley L. Scoping studies: towards a methodological framework. Int J Soc Res Methodol. 2005;8(1):19–32.

[29] Levac D, Colquhoun H, O’Brien K. Scoping studies: advancing the methodology. Implement Sci. 2010;5(1):69. 10.1186/1748-5908-5-69 PMC2954944 20854677

[30] Tricco A, Lillie E, Zarin W, O’Brien K, Colquhoun H, Levac D, et al. PRISMA Extension for Scoping Reviews (PRISMA-ScR): checklist and explanation. Ann Intern Med. 2018;169(7):467–73. 10.7326/M18-0850 30178033

[31] Braun V, Clarke V, Hayfield N, Terry G. Thematic Analysis. In: Liamputtong P, editor. Handbook of Research Methods in Health Social Sciences. Singapore: Springer; 2019. p. 843–60.

[32] Smith B & McGannon K. Developing rigor in qualitative research: problems and opportunities within sport and exercise psychology. International Review of Sport and Exercise Psychology. 2018; 11(1):101-121.

[33] Osinaike J, Hartley S (2021). Physical activity counselling among junior doctors in the UK: a qualitative study. Health Educ J.

[34] Sissons A, Grant A, Kirkland A, Currie S (2020). Using the theoretical domains framework to explore primary health care practitioner’s perspectives and experiences of preconception physical activity guidance and promotion. Psychol Health Med.

[35] Din N, Moore G, Murphy S, Wilkinson C, Williams N. Health professionals’ perspectives on exercise referral and physical activity promotion in primary care: findings from a process evaluation of the National Exercise Referral Scheme in Wales. Health Educ J. 2015;74(6):743–57. 10.1177/0017896914559785 PMC4604423 26527835

[36] Quirk H, Blake H, Dee B, Glazebrook C (2015). “Having diabetes shouldn’t stop them”: healthcare professionals’ perceptions of physical activity in children with type 1 diabetes. BMC Pediatr.

[37] McCourt O, Yong K, Ramdharry G, Fisher A (2021). Physical activity during and after haematological cancer treatment: a cross-sectional survey of haematology healthcare professionals in the united kingdom. J Multidiscip Healthc.

[38] Cunningham C, O’Sullivan R. Healthcare professionals’ application and integration of physical activity in routine practice with older adults: a qualitative study. Int J Environ Res Public Health. 2021;18(21):11222. 10.3390/ijerph182111222 PMC8582732 34769742

[39] Ward R, Miller P (2013). Depression, physical activity and mental health: an interpretative phenomenological analysis of general practitioners’ experiences of exercise referral schemes in the North West. Cumbria Partnersh J Res Pract Learn.

[40] Kime N, Pringle A, Zwolinsky S, Vishnubala D (2020). How prepared are healthcare professionals for delivering physical activity guidance to those with diabetes? A formative evaluation. BMC Health Serv Res.

[41] Buckley B, Finnie S, Murphy R, Watson P (2020). “You’ve got to pick your battles”: a mixed-methods investigation of physical activity counselling and referral within general practice. Int J Environ Res Public Health.

[42] Kassam H, Tzortziou Brown V, O’Halloran P, Wheeler P, Fairclough J, Maffulli N, et al. General practitioners’ attitude to sport and exercise medicine services: a questionnaire-based survey. Postgrad Med J. 2014;90(1070):680–4. 10.1136/postgradmedj-2013-132245 25352675

[43] Cottrell E, Roddy E, Rathod T, Porcheret M, Foster N. What influences general practitioners’ use of exercise for patients with chronic knee pain? Results from a national survey. BMC Fam Pract. 2016;17(1):172. 10.1186/s12875-016-0570-4 PMC5168590 27993126

[44] Wheeler P, Mitchell R, Ghaly M, Buxton K (2017). Primary care knowledge and beliefs about physical activity and health: a survey of primary healthcare team members. BJGP Open.

[45] Williams C, Gowing L, Horn R, Stuart AG (2017). A survey of exercise advice and recommendations in United Kingdom paediatric cardiac clinics. Cardiol Young.

[46] Fixsen D, Barrett D, Shimonovich M. Supporting vulnerable populations during the pandemic: stakeholders’ experiences and perceptions of social prescribing in Scotland during COVID-19. Qual Health Res. 2022;32(4):670–82. 10.1177/10497323211064229 PMC8948336 34969344

[47] Searle A, Calnan M, Turner K, Lawlor D, Campbell J, Chalder M (2012). General practitioners’ beliefs about physical activity for managing depression in primary care. Ment Health Phys Act.

[48] Avery L, Exley C, McPherson S, Trenell M, Anstee Q, Hallsworth K (2017). Lifestyle behavior change in patients with nonalcoholic fatty liver disease: a qualitative study of clinical practice. Clin Gastroenterol Hepatol.

[49] Speake H, Copeland R, Breckon J, Till S (2021). Challenges and opportunities for promoting physical activity in health care: a qualitative enquiry of stakeholder perspectives. Eur J Physiother.

[50] Shelley J, Fairclough SJ, Knowles Z, Southern K, McCormack P, Dawson E (2018). A formative study exploring perceptions of physical activity and physical activity monitoring among children and young people with cystic fibrosis and health care professionals. BMC Pediatr.

[51] Williamson C, Baker G, Mutrie N, Niven A, Kelly P (2020). Get the message? A scoping review of physical activity messaging. Int J Behav Nutr Phys Act.

[52] Graham R, Dugdill L, Cable N. Health professionals’ perspectives in exercise referral: implications for the referral process. Ergonomics. 2005;48(11–14):1411–22. 10.1080/00140130500101064 16338709

[53] Lawlor D, Keen S, Neal R. Increasing population levels of physical activity through primary care: GPs’ knowledge, attitudes and self-reported practice. Fam Pract. 1999;16(3):250–4. 10.1093/fampra/16.3.250 10439978

[54] Sallis R, Franklin B, Joy L, Ross R, Sabgir D, Stone J (2015). Strategies for promoting physical activity in clinical practice. Prog Cardiovasc Dis.

[55] Pugh G, O’Halloran P, Blakey L, Leaver H, Angioi M. Integrating physical activity promotion into UK medical school curricula: testing the feasibility of an educational tool developed by the faculty of sports and exercise medicine. BMJ Open Sport Exerc Med. 2020;6(1): e000679. 10.1136/bmjsem-2019-000679 PMC7279672 32547778

[56] Crozier A, Watson P, Graves L, George K, Naylor L, Green D (2022). Clinical exercise provision in the UK: comparison of staff job titles, roles and qualifications across five specialised exercise services. BMJ Open Sport Exerc Med.

[57] Tulloch H, Fortier M, Hogg W (2006). Physical activity counseling in primary care: who has and who should be counseling?. Patient Educ Couns.

[58] Cheema B, Robergs R, Askew C (2014). Exercise physiologists emerge as allied healthcare professionals in the era of non-communicable disease pandemics: a report from Australia, 2006–2012. Sports Med.

[59] Forsyth A, Deane F, Williams P. Dietitians and exercise physiologists in primary care: lifestyle interventions for patients with depression and/or anxiety. J Allied Health. 2009;38(2):63–8. 19753415

[60] McKenna J, Naylor P, McDowell N (1998). Barriers to physical activity promotion by general practitioners and practice nurses. Br J Sports Med.

[61] Reed B, Jensen J, Gorenflo D (1991). Physicians and exercise promotion. Am J Prev Med.

[62] Abramson S, Stein J, Schaufele M, Frates E, Rogan S (2000). Personal exercise habits and counseling practices of primary care physicians: a national survey. Clin J Sport Med.

[63] Crane E, Schaller G, Bergström M, Leivadiotou D, Simpson A (2021). How active are UK-based doctors?. Bullet Royal College Surg Engl.

[64] Michie S, van Stralen M, West R (2011). The behaviour change wheel: a new method for characterising and designing behaviour change interventions. Implement Sci.

